# Prognostic significance and oncogene function of cathepsin A in hepatocellular carcinoma

**DOI:** 10.1038/s41598-021-93998-9

**Published:** 2021-07-16

**Authors:** Huaxiang Wang, Fengfeng Xu, Fang Yang, Lizhi Lv, Yi Jiang

**Affiliations:** 1 The Fuzong Clinical Medical College of Fujian Medical University, Fuzhou, People’s Republic of China; 2 Department of Hepatobiliary Surgery, 900 Hospital of the Joint Logistic Team, No. 156 The Second West Ring Road, Fuzhou, 350025 Fu Jian Province People’s Republic of China

**Keywords:** Cancer, Genetics

## Abstract

Cathepsin A (CTSA) is a lysosomal protease that regulates galactoside metabolism. The previous study has shown CTSA is abnormally expressed in various types of cancer. However, rarely the previous study has addressed the role of CTSA in hepatocellular carcinoma (HCC) and its prognostic value. To study the clinical value and potential function of CTSA in HCC, datasets from the Cancer Genome Atlas (TCGA) database and a 136 HCC patient cohort were analyzed. CTSA expression was found to be significantly higher in HCC patients compared with normal liver tissues, which was supported by immunohistochemistry (IHC) validation. Both gene ontology (GO) and The Kyoto Encyclopedia of Genes and Genomes (KEGG) analyses demonstrated that CTSA co-expressed genes were involved in ATP hydrolysis coupled proton transport, carbohydrate metabolic process, lysosome organization, oxidative phosphorylation, other glycan degradation, etc. Survival analysis showed a significant reduction both in overall survival (OS) and recurrence-free survival (RFS) of patients with high CTSA expression from both the TCGA HCC cohort and 136 patients with the HCC cohort. Furthermore, CTSA overexpression has diagnostic value in distinguishing between HCC and normal liver tissue [Area under curve (AUC) = 0.864]. Moreover, Gene set enrichment analysis (GSEA) showed that CTSA expression correlated with the oxidative phosphorylation, proteasome, and lysosome, etc. in HCC tissues. These findings demonstrate that CTSA may as a potential diagnostic and prognostic biomarker in HCC.

## Introduction

Hepatocellular carcinoma (HCC) has a high mortality rate and is one of the cancers in which incidence gradually increased in recent years^1,2^. HCC causes a huge health burden globally, especially in East Asia and sub-Saharan Africa, and China accounts for about half of these deaths each year^3^. Although many researchers have made efforts to develop an effective therapy in recent decades, the 5-year survival has not significantly improved. Therefore, the identification of molecular biomarkers that can predict the prognosis of clinical treatment of HCC may substantially help the development of patient treatment strategies, and may even develop new targeted therapies for specific patients. The major risk factors for HCC are chronic infection with hepatitis B virus (HBV) and hepatitis C virus (HCV)^4,5^. Dietary aflatoxin exposure, chronic alcohol consumption, non-alcoholic fatty liver disease (NAFLD)/non-alcoholic steatohepatitis (NASH) are also linked to increased risk for HCC^3,6,7^. However, the complex signal transduction pathways and potential molecular pathogenesis involved in the occurrence and development of HCC are still unclear.

Cathepsin is a key lysosomal proteolytic enzyme, to the best of our knowledge, which is responsible for the degradation of many intra-cellular or extra-cellular substrates. Cathepsins were expressed in various types and stages of human cancer and were reported to be related to cancer progression and drug resistance^8–10^. Previous studies have found that the overexpression of cathepsin B leads to the invasiveness and metastasis of breast cancer, pancreatic cancer, HCC, and colorectal cancer, by activating the ErbB oncogenic signaling pathway^11–14^. The cytosol concentration of cathepsin D was found to have a strong influence on metastasis in breast cancer^15,16^. In addition, cathepsin K was found to have a high correlation with the progression of prostate cancer and breast cancer^17,18^. Cathepsin A (CTSA) is a renowned serine protease cathepsin member of the cathepsin lysosomal protease family, which with a role in protecting β-galactosidase and neuraminidase-1 from intra-lysosomal proteolysis^19^. In recent years, CTSA overexpression has been observed in various types of human cancers such as breast cancer, lung cancer, and prostate cancer^20–23^. CTSA also has been identified as a potential biomarker for early diagnosis, prognosis and monitoring during cancer treatment. However, the mechanism of action of CTSA in the development and progression of HCC has not been previously reported.

In this study, the gene expression profiling interactive analysis (GEPIA), The Cancer Genome Atlas (TCGA), The Human Protein Atlas databases were used to investigate the expression of CTSA in HCC and normal liver tissues to determine the relationship between prognosis of HCC and CTSA expression^24–26^. The relationship between the prognosis of HCC and CTSA expression was verified by Immunohistochemistry (IHC). The potential function of CTSA in HCC was analyzed by screening CTSA co-expressed genes using cBioPortal and LinkedOmics, as well as gene ontology (GO) and The Kyoto Encyclopedia of Genes and Genomes (KEGG) analyses performed using the Database for Annotation, Visualization and Integrated Discovery (DAVID)^27^. Then, we performed gene set enrichment analysis (GSEA) analysis to determine the enriched genes and whether a series of previously-defined 9 stages of HCC progress-related gene sets were enriched in different phenotypes^28^.

## Materials and methods

### Profiling of CTSA expression data

Genotype Tissue Expression (GTEx) projects and TCGA database provide mRNA expression data in various types and stages of human cancers. We used GEPIA (http://gepia.cancer-pku.cn/) which is a web server for analyzing the RNA-Seq expression data from the TCGA and GTEx projects to explore the expression distribution and correlation of CTSA in a body map and different cancer tissues. The GEPIA and the UCSC Xena project (https://xenabrowser.net/datapages/) has recomputed all raw expression data from TCGA database and were used to detect the CTSA expression in different stages and types of HCC^29^. Then, the Kaplan–Meier Plotter (http:// http://kmplot.com/analysis/) and GEPIA website to analyze the expression of CTSA and the prognosis of survival in HCC tissues^30^. The Human Protein Atlas database (https://www.proteinatlas.org/) were used to analyze the CTSA protein expression in HCC tissues and normal liver tissues by IHC. We used the Cancer Cell Line Encyclopedia project (CCLE) (https://portals.broadinstitute.org/ccle/) to analyze the relationship of CTSA mRNA levels with DNA copy number in different liver cancer cell lines. We obtained the information about the alteration of the CTSA gene in cBioPortal for Cancer Genomics (http://www.cbioportal.org/)^31^.

### Prognostic analysis using CTSA expression and clinicopathological data in HCC patients

We analyzed CTSA expression in HCC and adjacent peritumoral tissues from patients in TCGA database (https://xenabrowser.net/datapages/). We performed survival analysis to assess the clinical outcomes of patients with HCC after examination and transformation of variables evaluated in a Cox proportional hazards regression model. Overall survival (OS) was defined as the interval between surgery and mortality or between surgery and the last observation point. For surviving patients, the data were censored at the last follow-up. Recurrence-free survival (RFS) was defined as the interval between the date of surgery and the date of diagnosis of any type of relapse (intra-hepatic recurrence or extrahepatic metastasis)^32^. In order to evaluate the prognostic value of CTSA in HCC, 136 tumor specimens were collected during the continuous HCC tumors from September 2010 to December 2012, and the last follow-up was conducted on December 31, 2017. We used the Edmondson grading system^33^ and Child–Pugh classification to assess tumor differentiation and liver function, respectively. The 2010 International Union Against Cancer Tumor-Node-Metastasis (TNM) classification system were used to assess the tumor stages^34^.The patients must meet following criteria: liver function grade Child–Pugh class A, only one tumor lesion and absence of any metastasis, no cancer radiotherapy or chemotherapy prior to surgery, and pathology confirmed as primary HCC following surgery. The relevant clinicopathological data of HCC patients were obtained from the medical record of hospital database, and pathological data were assessed by two pathologists. Survival information was accessed from medical records, telephone interviews as well as the Social Security Death Index. This study was approved by the Human Research Ethics Committee of 900 Hospital of the Joint Logistics Team (Fuzhou, China). All experiments were performed in accordance with relevant named guidelines and regulations. All participants were supplied with written information and gave written consent prior to collection of the specimens and informed consent was obtained from all the participants.

### Immunohistochemistry (IHC) analyses

4-μm sections of 136 HCC tissue samples were fixed in super frost-charged glass microscope slides. Then, the tissue sections were deparaffinized and rehydrated using graded concentrations of malondialdehyde and ethanol. Antigen retrieval was performed by boiling sections in Tris/ Ethylenediaminetetraacetic acid (EDTA) (pH 9.0) for 20 min. Endogenous peroxidase was inhibited by incubation for 10 min in 3% H2O2 and washed in phosphate-buffered saline (PBS) three times. The sections were incubated with 10% normal goat serum for 30 min at room temperature (25 °C) without washing. A monoclonal rabbit Anti-CTSA antibody (1:300; 15,020–1-AP, Proteintech, Wuhan, China) were added dropwise to sections, incubated overnight at 4 °C, and washed in PBS three times. Then, sections were incubated with the secondary antibody (1:50,000; KIT-5010; anti-rabbit/mouse IgG; Maixin Biotechnology Development Co., Ltd., Fuzhou, China) at 37 °C for 30 min and were washed by PBS three times. Next, the sections were stained with 3,3′-diaminobenzidine and a substrate-chromogen (Dako) for 2 min at room temperature, and counterstained with hematoxylin for 40 s. Finally, the sections were dehydrated in 95% alcohol and sealed with neutral balsam.

### Evaluation of IHC staining

The sections were dropped by only the second antibody without the CTSA antibody was as the negative control. The 136 stained tissue sections were viewed using a CX41 microscope (Olympus, Tokyo, Japan) and assessed by two separate pathologists with no prior knowledge of any patient information. The expression of CTSA was predominantly cytoplasmic or cytomembrane based on the Human Protein Atlas database and previous studies^20,22^. A semi-quantitative IHC scoring system was used for the evaluation of CTSA protein level with a 5-point scale, as follows: 0, no positive cells; 1, < 25% positive cells; 2, 26–50% positive cells; 3, 51–75% positive cells; 4, > 75% positive cells. HCC tissue samples with a score of 0.1 or 2 were regarded as low CTSA expression, whereas a score of 3 or 4 was regarded as high CTSA expression.

### GO and KEGG enrichment analysis and PPI network construction

The hepatocellular carcinoma dataset in the cBioportal database and the LinkedOmics database were selected to analyze the correlated genes of CTSA expression using the function of co-expressed genes. Then, the overlapping genes with Pearson's Correlation greater than 0.35 obtained in the two databases were screened as CTSA co-expressed genes. Next, the Functional Annotation Tool in the DAVID database was used to perform GO and KEGG enrichment analysis on the co-expressed genes of CTSA^35,36^. In this process, the critical value of the significant gene functions and pathways to be screened was set as *P* < 0.05. Then, the protein–protein interaction (PPI) network of CTSA co-expressed genes was constructed in the STRING database, and the minimum required interaction score was set as 0.9(highest confidence)^37^. Finally, The Cytoscape software was used to visualize the PPI network^38^.

### GSEA enrichment analysis

Normalized gene expression data was downloaded in the TCGA database from the UCSC Xena database (https://xenabrowser.net/datapages/)^39^. Among them, there were 374 HCC specimens and 50 adjacent non-tumor tissue specimens. The 374 HCC specimens were divided into high expression group and low expression group taking the median of CTSA expression in HCC specimens as the critical point. We imported gene expression data into GSEA 4.1.0 software for enrichment analysis. In the process, we selected the KEGG gene sets (c2.cp.kegg.v7.0.symbols.gmt) as the functional gene set, the Number of permutations as 1000, and other parameters as the default settings. In the analysis of the results, the pathway of gene enrichment with a normal *p*-value < 0.05 and FDR q-value < 0.25 was selected.

### Genetic alteration of CTSA in HCC

We selected the Liver Hepatocellular Carcinoma (TCGA, Firehose Legacy) dataset in the cBioportal database to query the genetic alteration and the mutational hotspot of CTSA genes in HCC. Then, the overall survival rate was compared between HCC patients with CTSA mutations and without mutations.

### Statistical analysis

The statistical analysis was performed using Stata Statistical Software: SPSS 21 (SPSS Inc., Chicago, IL, USA) and GraphPad Prism 6.0 (GraphPad Software, Inc., San Diego, CA, USA). Pearson’s chi-square test was used to compare the categorical variables. For normally distributed continuous variables, the Student’s t-test was used. Survival was estimated using Kaplan–Meier plots with log-rank test for differences. The diagnostic significance of CTSA for HCC was evaluated by the receiver operating characteristic (ROC) curve. *P* < 0.05 was considered statistically significant unless otherwise stated.

## Results

### The relationship between the expression of CTSA and survival percentages of HCC patients in GEPIA and Kaplan–Meier plotters database

**Figure 1:**
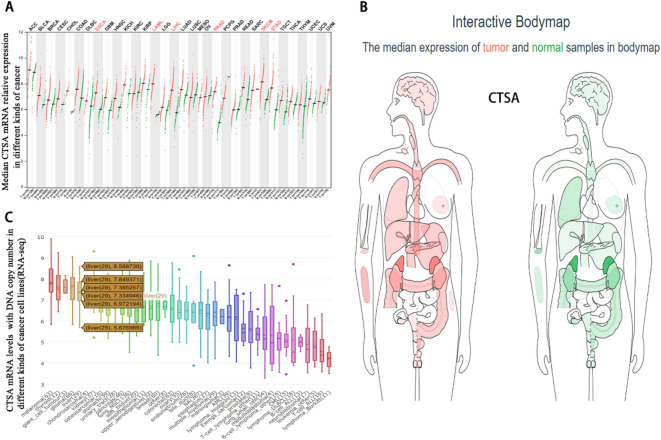
The mRNA expression level of CTSA distributed in different cancer tissues and in bodymap. (**A**) The CTSA expression profile across various kinds of tumor samples and paired normal tissues. Each dots represent the expression of tumor (red) and normal tissue (green). (**B**) The median CTSA expression of tumor (red) and normal samples (green) in bodymap in the GEPIA. (**C**) The CTSA mRNA levels with DNA copy number expressed in different kinds of cancer cell lines using the Cancer Cell Line Encyclopedia (CCLE) website. 29 HCC cell lines have variations in CTSA expression.

**Figure 2:**
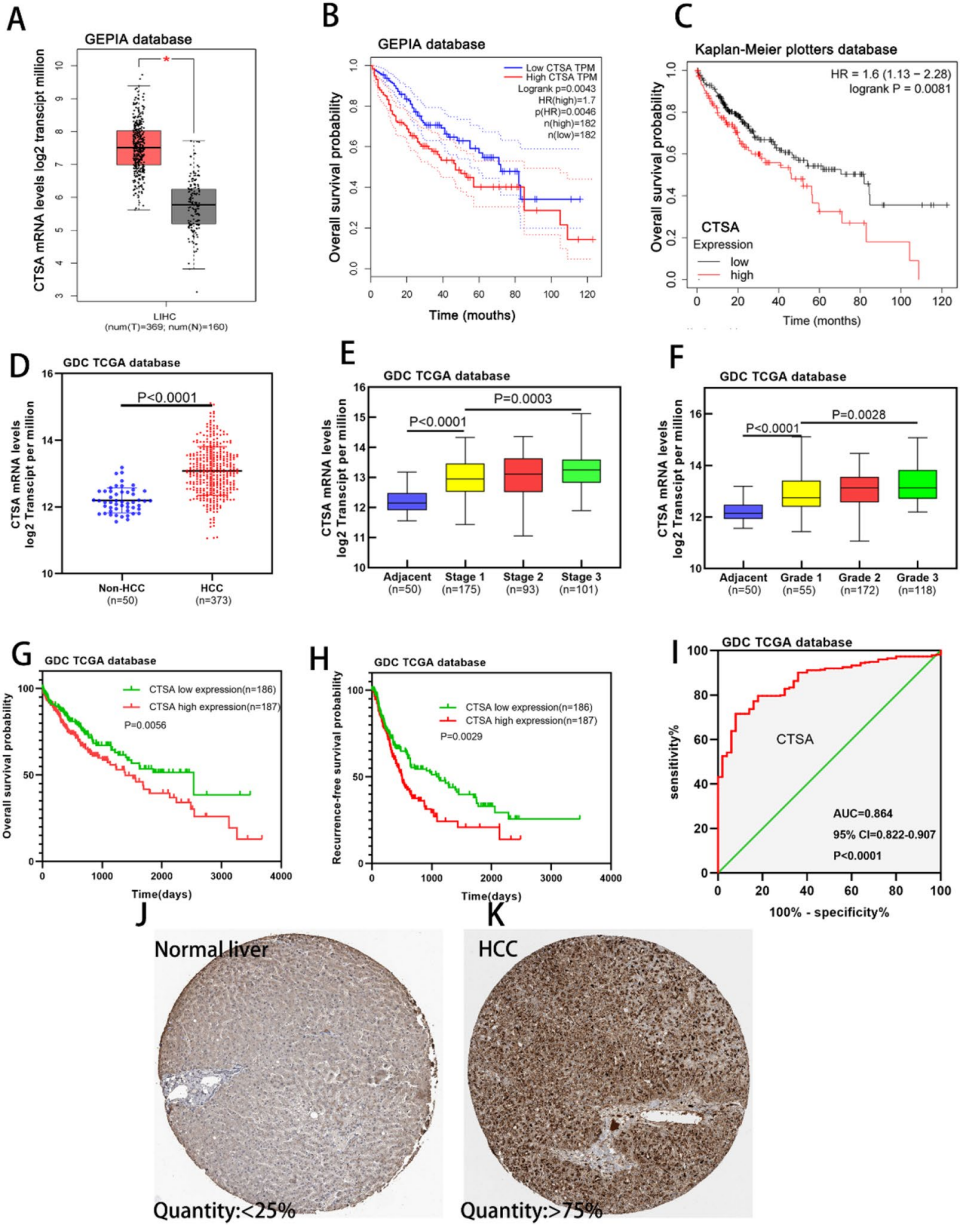
Analysis of CTSA expression and prognostic value of HCC patients in the TCGA database. (**A**) The CTSA expression level in hepatocellular carcinoma was significantly higher than normal liver tissues in the GEPIA database (*P* < 0.05). (**B**,**C**) High CTSA expression level was associated with poor overall survival (OS) in patients with HCC in the GEPIA website and Kaplan–Meier plotters database. (**D**) The CTSA expression level in HCC was significantly higher than None-HCC tissues in the TCGA database (*P* < 0.0001). (**E**,**F**) The CTSA expression in HCC was incrementally upregulated with increasing tumor stages and neoplasm histology grades in the TCGA database. (**G**,**H**) Kaplan–Meier curves of overall survival (OS) and recurrence-free survival (RFS) among different CTSA expression among HCC patients in the TCGA database. High CTSA expression associated with poor OS (G, *P* = 0.0056) and RFS (**H**, *P* = 0.0029). (**I**) The receiver operating characteristic (ROC) curve showed that the CTSA expression level can be used as evidence to predict the prognosis of HCC patients (AUC = 0.864, *P* < 0.0001). (**J**,**K**) Representative images in the Human Protein Atlas database showed that the expression quantity of CTSA protein in HCC tissues (> 75%, **K**) was higher than normal liver tissues (< 25%, **J**).

We found that the expression of CTSA mRNA in a variety of tumor tissues was significantly higher than normal tissues in the GEPIA database (Fig. 1A). Besides, the human body map shows that the mRNA expression level of CTSA is significantly up-regulated in liver cancer tissues compared with normal liver (Fig. 1B). We analyzed the CCLE database and found that the CTSA mRNA expression copy number is significantly different in various kinds of cancer cell lines (Fig. 1C). In the GEPIA database, CTSA is the significantly high expression in HCC patients (Fig. 2A) and which is associated with a poor prognosis (Fig. 2B). We evaluated the relationship between the CTSA mRNA levels and overall survival in HCC patients using the Kaplan–Meier plotters database and found that the high expression cohort had a shorter median survival time (*P* = 0.0081) (Fig. 2C).

### The relationship between the expression of CTSA and clinical outcomes in HCC patients in TCGA database

We downloaded the HCC dataset from the TCGA database to analyze the relationship between CTSA mRNA expression and the clinical outcome of HCC patients. The mRNA expression of CTSA in the HCC group (n = 373) was significantly higher than in the none-HCC group (n = 50) (*P* < 0.001, Fig. 2D). The expression level of CTSA was significantly positively correlated with the TNM staging of HCC (Fig. 2E). In addition, the mRNA expression of CTSA was incrementally upregulated with increasing neoplasm histology grades (Fig. 2F). The high expression of CTSA mRNA was related to the vascular invasion (*P* = 0.001), tumor TNM stage (*P* = 0.004), serum alpha-fetoprotein (AFP) level (*P* = 0.001), neoplasm histology grades (*P* = 0.038), and adjacent hepatic inflammation in (*P* = 0.009) HCC patients. Age, gender, family cancer history, preoperative radiotherapy, preoperative pharmaceutical, and body mass index (BMI) were not related to CTSA mRNA expression (Table 1). We found that vascular invasion (*P* = 0.014), tumor TNM staging (*P* < 0.001), and high expression of CTSA mRNA (*P* = 0.006) were risk factors of overall survival by performing Univariate Cox Regression analysis. The Multivariate Cox Regression analysis confirmed that TNM staging (HR (95%CI) 0.492 (0.346–0.700); *P* = 0.001) and high CTSA mRNA expression (HR (95%CI) 1.420 (1.001–2.013); *P* = 0.049) were independent risk factors for overall survival. For RFS, the vascular invasion (*P* < 0.001), tumor TNM staging (*P* < 0.001), Serum AFP level (*P* = 0.029), and high expression of CTSA mRNA (*P* = 0.003) were risk factors. Meanwhile, the vascular invasion (HR (95%CI) 1.651 (1.189–2.291); *P* = 0.003), TNM staging (HR (95%CI) 0.653 (0.465–0.919); *P* = 0.014) were independent risk factors of RFS confirmed by Multivariate Cox Regression (Table 2). As shown in the Kaplan–Meier curve, the overall survival OS (*P* = 0.0056; Fig. 2G) percentage and RFS (*P* = 0.0029; Fig. 2H) percentage of patients with high CTSA expression are poorer than low CTSA expression patients. The median OS time of the CTSA mRNA high expression cohort and low expression cohort was 1836 days and 2456 days, and the median RFS time of the two cohorts was 498 and 1117 days, respectively. The ROC curve showed that the expression level of CTSA mRNA can be used as evidence to predict the prognosis of HCC patients (Area under curve (AUC) = 0.864, *P* < 0.0001, Fig. 2I). Furthermore, we analyzed the expression level of CTSA protein in the Human Protein Atlas database (https://www.proteinatlas.org/) and found that its expression quantity in HCC tissues (> 75%, Fig. 2J) was higher than normal liver tissues (< 25%, Fig. 2K).

**Table 1:** Correlation between CTSA expression and clinical outcomes in HCC patients (373 cases, TCGA database).

Characteristics	CTSA level			
	N	High (n)	Low (n)	*P*-value*
Gender				
Male	251	130	121	0.358
Female	122	57	65	
Age (years)				
> 55	246	127	119	0.423
< = 55	127	60	67	
Vascular invasion				
Yes	139	84	53	0.001
No	234	103	133	
TNM staging				
I/II	268	122	146	0.004
III/IV	105	65	40	
Serum AFP level (ng/ml)				
> 400 ng/ml	133	82	51	0.001
< = 400 ng/ml	240	105	135	
Neoplasm histology grades				
G1/G2	227	104	123	0.038
G3/G4	146	83	63	
Famliy cancer history				
Yes	115	65	50	0.068
No	209	96	113	
Adjancent hepatic inflammation				
Yes	174	73	101	0.009
No	154	87	67	
Radiation				
Yes	22	15	7	0.081
No	341	172	169	
Pharmaceutical				
Yes	42	25	17	0.196
No	331	162	169	
BMI (kg/m^2^)				
> = 24	178	95	83	0.396
< 24	158	77	81

**Table 2:** Univariate and Multivariate Cox Regression analysis of overall survival and Recurrence-free survival in HCC patients (373 cases, TCGA database).

Variables	Overall survival	*P*-Value*	Recurrence-free survival	*P*-Value*
	HR (95%CI)		HR (95%CI)	
Univariate analysis				
Gender				
Male vs. female	0.823 (0.530–1.168)	0.275	0.961 (0.707–1.307)	0.800
Age (years)				
> 55 vs. < = 55	1.197 (0.832–1.721)	0.333	0.924 (0.681–1.253)	0.611
Vascular invasion				
Yes vs. no	1.531 (1.090–2.151)	0.014	1.843 (1.373–2.474)	< 0.001
TNM staging				
I/II vs. III/IV	0.448 (0.318–0.632)	< 0.001	0.547 (0.374–0.688)	< 0.001
Serum AFP level (ng/ml)				
> 400 vs < = 400	1.351 (0.956–1.908)	0.088	1.399 (1.036–1.890)	0.029
Neoplasm histology grades				
G1/G2 vs. G3/G4	0.958 (0.673–1.363)	0.810	0.914 (0.674–1.236)	0.559
Famliy cancer history				
Yes vs. no	1.105 (0.768–1.590)	0.590	0.846 (0.610–1.173)	0.316
Adjancent hepatic inflammation				
Yes vs. no	0.981 (0.675–1.426)	0.919	1.115 (0.818–1.519)	0.491
Radiation				
Yes vs. no	1.007 (0.492–2.060)	0.985	1.160 (0.645–2.087)	0.620
Pharmaceutical				
Yes vs. no	0.895 (0.537–1.490)	0.669	0.829 (0.539–1.274)	0.391
BMI (kg/m^2^)				
> = 24 vs. < 24	0.700 (0.485–1.008)	0.055	0.807 (0.594–1.097)	0.172
CTSA				
High vs. low	1.165 (1.147–2.274)	0.006	1.564 (1.162–2.104)	0.003
Multivariate analysis				
Vascular invasion				
Yes vs. no	1.421 (0.968–2.085)	0.073	1.651 (1.189–2.291)	0.003
TNM staging				
I/II vs. III/IV	0.492 (0.346–0.700)	0.001	0.653 (0.465–0.919)	0.014
Serum AFP level (ng/ml)				
> 400 vs < = 400	1.094 (0.742–1.613)	0.651	1.224 (0.876–1.711)	0.236
CTSA				
High vs. low	1.420 (1.001–2.013)	0.049	1.347 (0.994–1.824)	0.054

*HR* hazard ratio, *CI* confidential interval, *TNM* tumor, node, metastasis, *AFP* alpha fetoprotein, *BMI* body mass index, *CTSA* Cathepsin A.

*P-values < 0.05 were considered statistically significant.

### The relationship between the CTSA protein expression and clinicopathologic characteristics in 136 HCC patients

**Figure 3:**
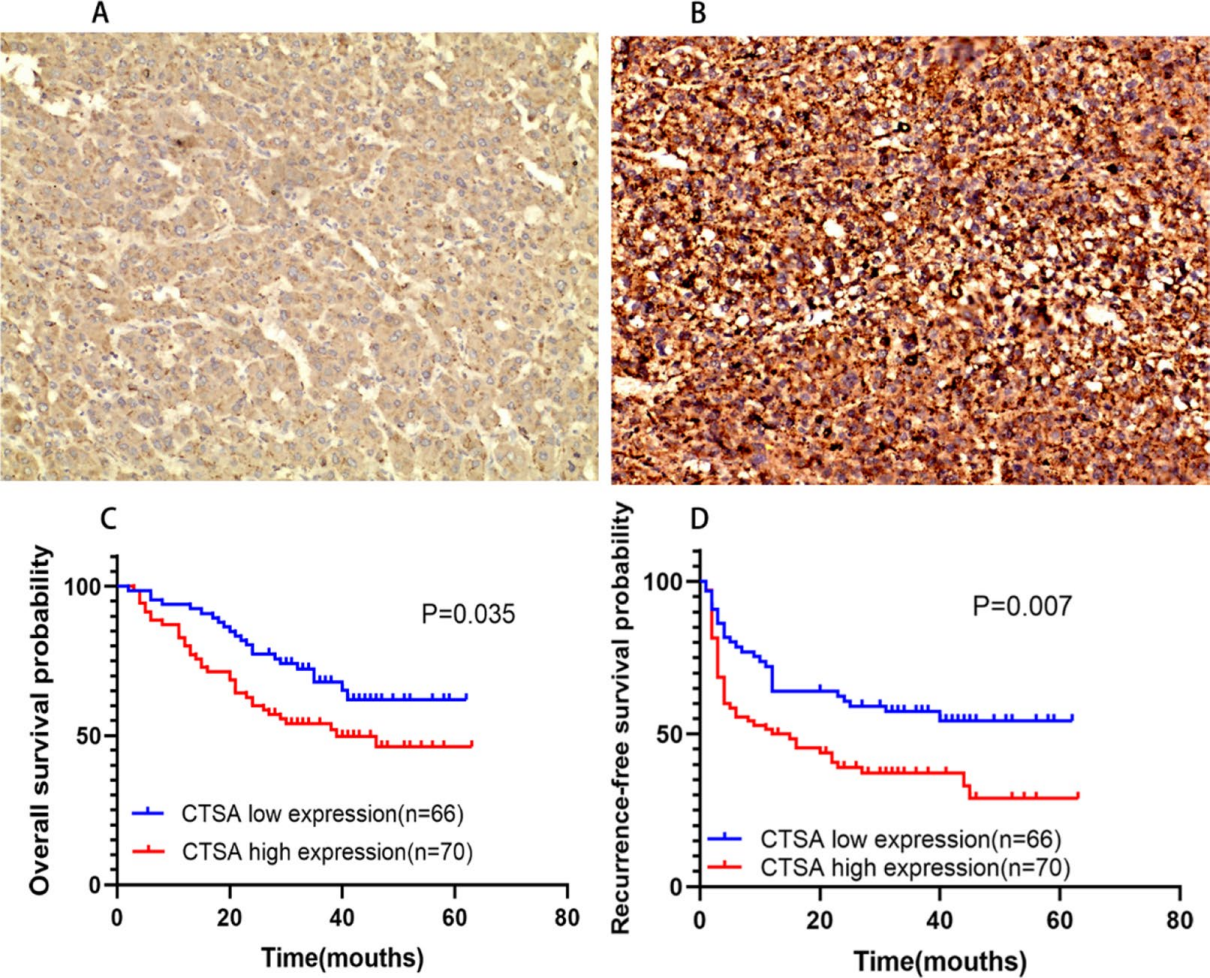
Analysis of CTSA expression and prognostic value of 136 cases of HCC patients. (**A**,**B**) Representative image of CTSA low (**A**)/high (**B**) expression in tumor tissue from patients with HCC (× 200 magnification). (**C**,**D**) Kaplan–Meier curves of overall survival (OS) and recurrence-free survival (RFS) among different CTSA expression among 136 cases of HCC patients. High CTSA expression associated with poor OS (**G**, *P* = 0.0035) and RFS (**H**, *P* = 0.007).

As shown in the figure, CTSA mainly expressed on the cytoplasm and cell membrane of HCC (Fig. 3A,B). According to the A semi-quantitative IHC scoring system, 70 of 136 patients showed high CTSA expression and 66 had a low expression. The high CTSA protein expression of HCC patients was related to TNM staging (*P* = 0.024), serum AFP level (*P* = 0.001), tumor location (*P* = 0.037), tumor differentiation (*P* = 0.031), tumor recurrence (*P* = 0.013), and survival (*P* = 0.036), but not related to age, gender, tumor size, vascular invasion, and tumor encapsulation (Table 3). The Univariate Cox Regression analysis showed that tumor size (*P* = 0.033), TNM stage (*P* = 0.005), serum AFP level (*P* = 0.036), tumor differentiation (*P* = 0.008), vascular invasion (*P* = 0.048), and high expression of CTSA (*P* = 0.039) were risk factors affecting OS. The Multivariate analysis confirmed that tumor differentiation (HR (95%CI) 3.590 (1.093–11.795); *P* = 0.035) and high CTSA expression (HR (95%CI) 0.575 (0.336–0.983); *P* = 0.043) are independent risk factors affecting OS. For RFS, tumor differentiation (HR (95%CI) 2.938 (1.161–7.433); *P* = 0.023), vascular invasion (HR (95%CI) 2.075 (1.271–3.390); *P* = 0.004), tumor non-encapsulation (HR (95%CI) 0.288 (0.177–0.467); *P* < 0.001), and high CTSA expression (HR (95%CI) 0.583 (0.362–0.939); *P* = 0.027) were independent risk factors confirmed by the Multivariate analysis (Table 4). As shown in the Kaplan–Meier curve, the overall survival OS (*P* = 0.0035; Fig. 3C) percentage and RFS (*P* = 0.0007; Fig. 3D) percentage of patients with high CTSA expression are poorer than low CTSA expression patients. The median OS time of the CTSA protein high expression cohort and low expression cohort was 39 mouths and 60 mouths, and the median RFS time of the two cohorts was 12 mouths and 40 mouths, respectively.

**Table 3:** Correlation between CTSA expression and clinical outcomes in HCC patients (n = 136).

Characteristics	CTSA level			
	N	High (n)	Low (n)	*P*-value*
Age (year)				
> 55	70	48	42	0.589
< = 55	66	22	24	
Gender				
Male	119	59	60	0.304
Female	17	11	6	
Tumor size (cm)				
> 5 cm	71	37	34	0.506
< = 5 cm	65	33	32	
TNM staging				
I/II	88	39	49	0.024
III	48	31	17	
Serum AFP level				
> 400 ng/ml	63	42	21	0.001
< = 400 ng/ml	73	28	45	
Tumor location				
Left	44	28	16	0.037
Right	92	42	50	
Tumor differentiation				
Low	14	9	5	0.031
Median	97	53	44	
High	25	8	17	
Vascular invasion				
Yes	68	36	32	0.432
No	68	34	34	
Tumor encapsulation				
Yes	51	42	48	0.083
No	191	28	18	
Recurrence				
Yes	74	45	29	0.013
No	62	25	37	
Survival				
Alive	79	35	44	0.036
Dead	57	35	22

**Table 4:** Univariate and multivariate cox regression analysis of overall survival and recurrence-free survivalin in HCC patients (n = 136).

Variables	Overall survival	*P*-Value*	Recurrence-free survival	*P*-Value*
	HR (95%CI)		HR (95%CI)	
**Univariate analysis**				
Age (year)				
> 55 vs. < = 55	0.793 (0.465–1.352)	0.394	0.703 (0.437–1.131)	0.146
Gender				
Male vs. female	1.219 (0.523–2.842)	0.646	1.403 (0.738–2.665)	0.301
Tumor size (cm)				
> 5 vs. < = 5	1.811 (1.048–3.130)	0.033	1.592 (0.993–2.551)	0.053
TNM staging				
I/II vs. III	2.092 (1.244–3.518)	0.005	1.438 (0.897–2.304)	0.131
Serum AFP level				
> 400 vs < = 400	1.753 (1.038–2.961)	0.036	1.350 (0.853–2.138)	0.201
Tumor location				
Left vs. right	0.797 (0.462–1.373)	0.413	1.142 (0.693–1.884)	0.602
Tumor differentiation				
Hihg vs. median/low	4.788 (1.496–15.320)	0.008	4.132 (1.663–10.262)	0.002
Vascular invasion				
Yes vs. no	1.707 (1.005–2.901)	0.048	2.369 (1.462–3.839)	< 0.001
Tumor encapsulation				
Yes vs. no	1.776 (0.455–1.324)	0.353	0.258 (0.161–0.416)	< 0.001
CTSA				
High vs. low	0.569 (0.334–0.971)	0.039	0.537 (0.334–0.862)	0.010
**Multivariate analysis**				
Tumor size (cm)				
> 5 vs. < = 5	1.142 (0.550–2.373)	0.722		
TNM staging				
I/II vs. III	1.622 (0.931–2.825)	0.088		
Serum AFP level				
> 400 vs < = 400	1.212 (0.696–2.110)	0.498		
Tumor differentiation				
Hihg vs. median/low	3.590 (1.093–11.795)	0.035	2.938 (1.161–7.433)	0.023
Vascular invasion				
Yes vs. no	1.306 (0.753–2.263)	0.342	2.075 (1.271–3.390)	0.004
Tumor encapsulation				
Yes vs. no			0.288 (0.177–0.467)	< 0.001
CTSA				
High vs. low	0.575 (0.336–0.983)	0.043	0.583 (0.362–0.939)	0.027

### Analysis of CTSA alteration using RNA-sequencing data set in cBioPortal database

**Figure 4:**
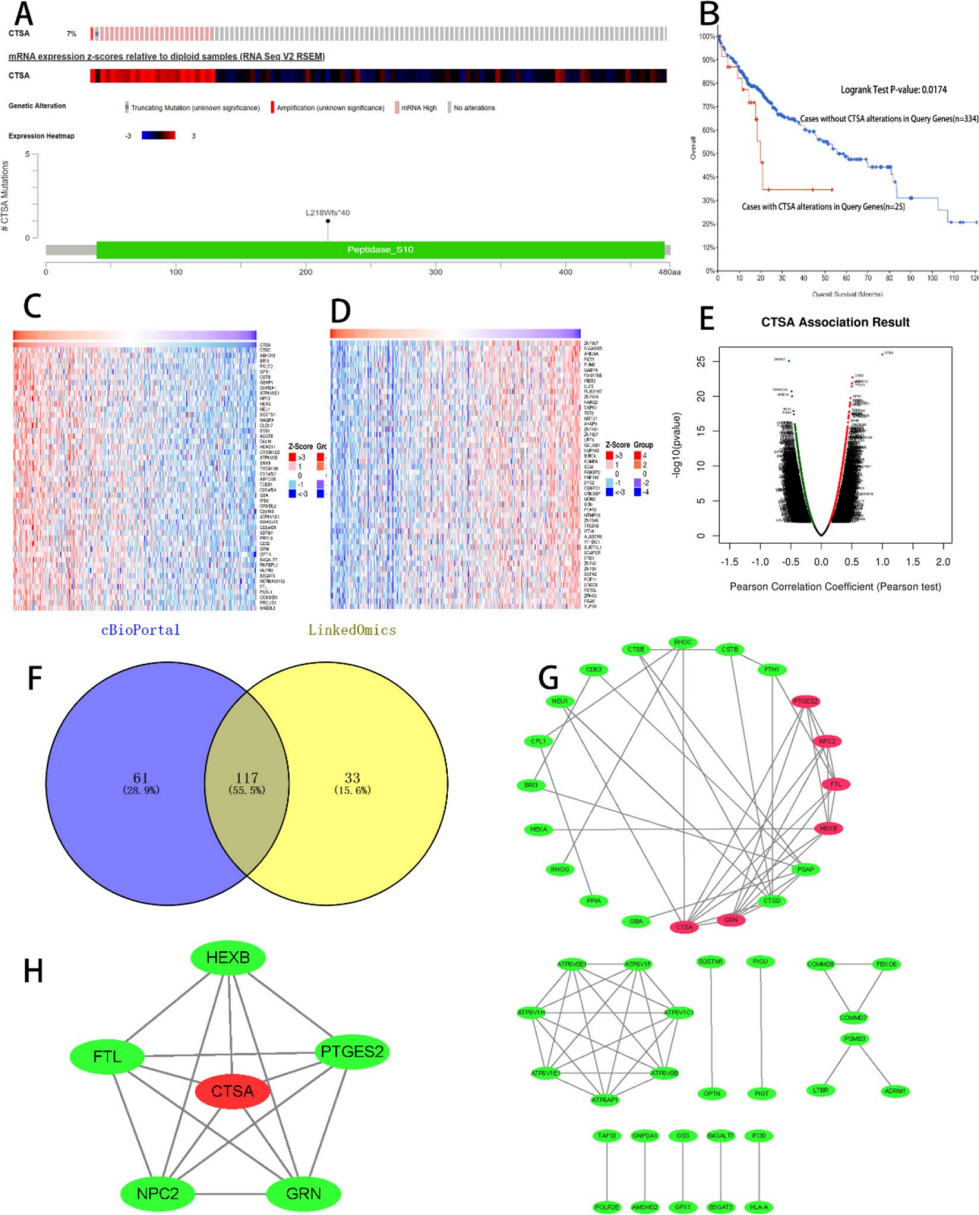
Alteration analyzed in RNA-sequencing data and identification of CTSA co-expressed genes. (**A**) The alteration and Expression Heatmap of CTSA in a cohort of 359 HCC patients (TCGA, Firehose Legacy) from the cBioPortal database. 7% queried genes in this cohort exhibited alteration. One mutational hotspot, L218Wfs*40/frameshift deletion mutation was labeled. (**B**) the Kaplan–Meier curve shows that the OS percentage of HCC patients with CTSA alterations (n = 25) is poorer than without CTSA alterations. (**C**–**E**) CTSA correlated target genes analysis in the linkedomics database. (**C**) The top 50 genes that are positively correlated to CTSA. (**D**) The top 50 genes that are negatively correlated to CTSA. (**E**) Volcano chart display CTSA Positively/Negatively Correlated Significant Genes. (**F**) 117 overlapping genes with Spearman's Correlation greater than 0.35 obtained in the cBioportal database and the LinkedOmics database were screened as co-expressed genes of CTSA. (**G**) The 117 co-expressed genes of CTSA were used to construct the PPI networks with a confidence score of > 0.900 (highest confidence). (**H**) The PPI network showed that the FTL, GRN, NPC2, HEXB, and PTGES2 protein can interact with CTSA.

we queried the genetic alterations of CTSA in a cohort of 359 HCC patients (TCGA, Firehose Legacy) in the cBioportal database and found that queried gene is altered in 25 (7%) of queried HCC patients, including 1 case of truncating mutation, 1 case of amplification, and 23 cases of mRNA high expression. We also analyzed the mutational hotspot of CTSA in 359 HCC patients and found that there was one mutational hotspot, L218Wfs*40/frameshift deletion mutation (Fig. 4A). The percentage of samples with a somatic mutation in CTSA is 0.3%. In addition, the Kaplan–Meier curve shows that the OS percentage of LIHC patients with CTSA alterations (n = 25) is poorer than without CTSA alterations (n = 334) (*P* = 0.0174, Fig. 4B).

### GO and KEGG enrichment analysis and PPI network construction of CTSA co-expressed genes

**Figure 5:**
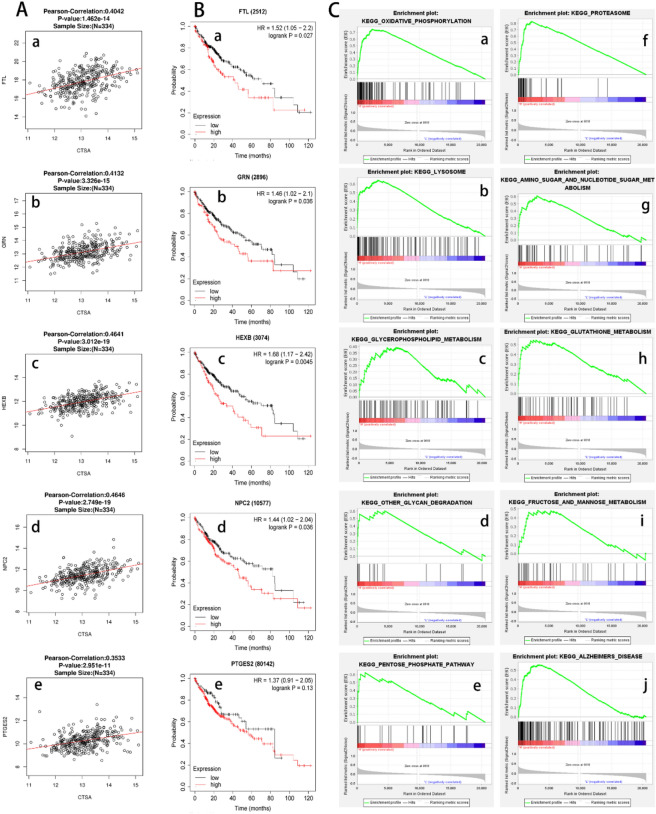
(**A**) Correlation between CTSA and FTL (*r* = 0.4042), GRN (*r* = 0.4132), HEXB (0.4641), NPC2, (*r* = 0.4646), PTGES2 (*r* = 0.3533), all *P* < 0.05. (**B**) The Kaplan–Meier curve showed that the mRNA expression of FTL, GRN, NPC2, and HEXB were significantly related to the OS in HCC patients (all *P* < 0.05). (**C**) The main enriched KEGG pathways of CTSA. OXIDATIVE PHOSPHORYLATION, LYSOSOME, OTHER GLYCAN DEGRADATION, PENTOSE PHOSPHATE PATHWAY, PROTEASOME, AMINO SUGAR AND NUCLEOTIDE SUGAR METABOLISM, GLUTATHIONE METABOLISM, FRUCTOSE AND MANNOSE METABOLISM, ALZHEIMERS DISEASE, GLYCEROPHOSPHOLIPID METABOLISM.

We selected the liver hepatocellular carcinoma dataset in the cBioportal database and the LinkedOmics database to analyze the correlated genes of CTSA expression using the function of co-expressed genes. A total of 19,899 genes related to CTSA protein expression in a cohort of 371 HCC patients from the LinkedOmics database expression were investigated. We exhibited a gene heat map and volcano plot with 8846 positively and 11,053 negatively correlated genes with CTSA protein expression (Fig. 4C–E). A total of 117 overlapping genes with Spearman's Correlation greater than 0.35 obtained in the cBioportal database and the LinkedOmics database were screened as co-expressed genes of CTSA (Fig. 4F). Next, we performed GO and KEGG enrichment analysis for 117 co-expressed genes of CTSA using DAVID 6.8 to predict the potential function and pathway of CTSA in HCC. The GO analysis showed that 117 cases of co-expressed genes of CTSA were mainly enriched in biological processes, such as ATP hydrolysis coupled proton transport, Carbohydrate metabolic process, Ganglioside catabolic process, Lysosome organization, and Negative regulation of fibroblast proliferation, etc. The KEGG enrichment analysis showed that 117 co-expressed genes of CTSA were mainly enriched in the signal pathway of Lysosome, Oxidative phosphorylation, Metabolic pathways, Amino sugar, and nucleotide sugar metabolism, etc. (Table 5). The 117 co-expressed genes of CTSA were analyzed in the STRING database to identify the significant interactions and were visualized using Cytoscape software. 47 nodes with 70 edges were selected to construct the PPI networks with a confidence score of > 0.900 (highest confidence) (Fig. 4G). The PPI network showed that the FTL, GRN, NPC2, HEXB, and PTGES2 protein can interact with CTSA (Fig. 4H). The Pearman correlation test analysis in the LinkedOmics database confirmed that the protein expression of FTL, GRN, NPC2, HEXB, and PTGES2 significantly positively correlated with CTSA (FTL: *r* = 0.4042, GRN: *r* = 0.4132, HEXB: 0.4641, NPC2: *r* = 0.4646, PTGES2: *r* = 0.3533, all *P* < 0.05) (Fig. 5A). We also performed the Kaplan–Meier curve and log-rank test analyses in the Kaplan–Meier plotters database and the result showed that the mRNA expression of FTL, GRN, NPC2, and HEXB were significantly related to the OS in HCC patients (all *P* < 0.05) (Fig. 5B).

**Table 5:** The main GO and KEGG pathway enrichment analysis for 117 co-expressed genes.

Category	ID term	Term	Count	*P* value	Benjamini	FDR
GOTERM_Biological Process	GO:0015991	ATP hydrolysis coupled proton transport	7	5.78E−08	2.32E−05	2.31E−05
GOTERM_Biological Process	GO:0005975	Carbohydrate metabolic process	6	5.19E−04	6.61E−02	6.58E−02
GOTERM_Biological Process	GO:0006689	Ganglioside catabolic process	3	6.93E−04	6.61E−02	6.58E−02
GOTERM_Biological Process	GO:0006880	Intracellular sequestering of iron ion	3	6.93E−04	6.61E−02	6.58E−02
GOTERM_Biological Process	GO:0007040	Lysosome organization	4	8.22E−04	6.61E−02	6.58E−02
GOTERM_Biological Process	GO:0006826	Iron ion transport	3	2.48E−03	1.66E−01	1.66E−01
GOTERM_Biological Process	GO:0048147	Negative regulation of fibroblast proliferation	3	8.24E−03	4.73E−01	4.71E−01
GOTERM_Biological Process	GO:0006897	Endocytosis	4	1.29E−02	6.50E−01	6.46E−01
KEGG_PATHWAY	hsa04142	Lysosome	16	2.45E−14	2.72E−12	2.59E−12
KEGG_PATHWAY	hsa04721	Synaptic vesicle cycle	7	1.58E−05	8.75E−04	8.35E−04
KEGG_PATHWAY	hsa01100	Metabolic pathways	24	5.73E−05	1.81E−03	1.73E−03
KEGG_PATHWAY	hsa05323	Rheumatoid arthritis	7	6.53E−05	1.81E−03	1.73E−03
KEGG_PATHWAY	hsa00511	Other glycan degradation	4	5.29E−04	1.17E−02	1.12E−02
KEGG_PATHWAY	hsa00520	Amino sugar and nucleotide sugar metabolism	5	7.80E−04	1.44E−02	1.38E−02
KEGG_PATHWAY	hsa04145	Phagosome	7	1.03E−03	1.48E−02	1.41E−02
KEGG_PATHWAY	hsa00190	Oxidative phosphorylation	7	1.07E−03	1.48E−02	1.41E−02

### GSEA enrichment analysis of CTSA expression in TCGA database

We performed GSEA enrichment analysis in the GSEA 4.1.0 software to investigate the potential pathway that CTSA may regulate the carcinogenesis and development of HCC using normalized gene expression data in the TCGA HCC dataset downloaded from the UCSC Xena database. The GSEA revealed that KEGG pathway associated with carcinogenesis and development including "oxidative phosphorylation", "proteasome", "lysosome", "glycerophospholipid metabolism", "other glycan degradation", "pentose phosphate pathway", and "amino sugar and nucleotide sugar metabolism" were identified as significantly altered in CTSA high group (Fig. 5C). Furthermore, the result showed that the genes involving in these KEGG pathways were significantly altered in the high CTSA expression group (Table 6). In summary, we considered that CTSA regulated the occurrence and development of HCC may through these signaling pathways.

**Table 6:** The main enriched KEGG pathways of CTSA highexpression in GDC TCGA liver cancer cohort.

KEGG enrichment signal pathway	ES	NES	NOM *p*-value	FDR q-value
KEGG_OXIDATIVE_PHOSPHORYLATION	0.750	1.990	0.000	0.009
KEGG_LYSOSOME	0.640	2.370	0.000	0.000
KEGG_OTHER_GLYCAN_DEGRADATION	0.600	1.620	0.043	0.208
KEGG_PENTOSE_PHOSPHATE_PATHWAY	0.620	1.910	0.002	0.021
KEGG_PROTEASOME	0.830	1.950	0.002	0.012
KEGG_AMINO_SUGAR_AND_NUCLEOTIDE_SUGAR_METABOLISM	0.610	2.000	0.000	0.008
KEGG_GLUTATHIONE_METABOLISM	0.550	1.790	0.010	0.064
KEGG_FRUCTOSE_AND_MANNOSE_METABOLISM	0.480	1.650	0.018	0.184
KEGG_ALZHEIMERS_DISEASE	0.560	2.010	0.000	0.014
KEGG_GLYCEROPHOSPHOLIPID_METABOLISM	0.390	1.520	0.025	0.301

*ES* enrichment score, *NES* normalized enrichment score, *FDR* false discovery rate.

## Discussion

HCC was a highly aggressive malignant tumor with high mortality and caused a huge health burden globally. According to data in 2019 released by the American Cancer Society^40^, the 5-year survival percentage of HCC patients for all stages was only 18%, and cancer-related mortality ranks 5th among all cancers. Even though the methods of diagnosis and treatment have got rapid development, HCC still lacks effective diagnostic biomarkers. In the past few years, the role of various classes of cathepsins in the proliferation and metastasis of various types of human cancers has been extensively studied. The overexpression of cathepsin B promotes invasion and metastasis of breast cancer, pancreatic cancer, HCC, and colorectal cancer^11–14^. In addition, cathepsin B has been found to be involved in tumor initiation, migration, and drug resistance of glioblastoma stem cells and prostate cancer stem cells^41,42^. CTSA is a well-known serine protease cathepsin member of the cathepsin lysosomal protease family, which has been identified as a potential biomarker for early diagnosis, prognosis, and monitoring during cancer treatment^20,21,43^. A previous study showed that knockdown of CTSA suppressed the metastasis of prostate cancer by reducing the phosphorylation of the P38 MAPK pathway^22^. Another previous study had found that CTSA is highly expressed in hepatocellular carcinoma through the method of quantitative proteomics^44^, but its clinical prognostic value and gene function never been illustrated. This study is the first systematic investigation of diagnostic value, clinical significance, and the gene function of CTSA in HCC.

We analyzed the mRNA expression level of CTSA using the GEPIA database and TCGA database and found that its expression in HCC tissues was significantly higher than adjacent tissues. And this result was confirmed by IHC from the Human Protein Atlas database and 136 cases of clinical specimens. Furthermore, the high-level expression of CTSA mRNA was significantly correlated with poor OS and PFS of HCC patients in the GEPIA database, Kaplan–Meier plotters database, and TCGA database as well as the 136 HCC patients, indicating that CTSA may perform an important role in the development of HCC. To further investigate the clinical significance of CTSA in HCC, we analyzed the relationship between clinicopathological variables records and CTSA expression from the TCGA database and 136 HCC patients and found that the high mRNA expression of CTSA was significantly associated with vascular invasion, TNM staging, serum AFP level, neoplasm histology grades, adjacent hepatic inflammation, tumor recurrence, and survival. And the multivariate regression analysis confirmed that the high mRNA expression of CTSA was an independent risk factor for OS in HCC patients. High protein expression of CTSA was an independent risk factor for OS and RFS in HCC patients (Table 4). Previous studies had shown that CTSA can be used as a biomarker of the prognostic value of HCC^44^. Our research was consistent with it. The ROC curve using data from the TCGA database indicating that CTSA mRNA expression has a significant diagnostic value between HCC and normal liver tissues. IHC is a routine pathological examination after HCC resection. Our research showed that CTSA protein expression was significantly increased in HCC and was an independent risk factor for OS and RFS. Therefore, postoperative CTSA IHC examination can help predict the recurrence and prognosis of HCC patients.

In order to explore the function of CTSA in the process of tumorigenesis and development of HCC, we identified the co-expressed genes using cBioPortal and LinkedOmic databases, and then performed GO and KEGG enrichment analysis used DAVID software on co-expressed genes. The results show that CTSA mainly involves in biological processes, such as ATP hydrolysis coupled proton transport, the Carbohydrate metabolic process, Ganglioside catabolic process, Lysosome organization, and Negative regulation of fibroblast proliferation, etc. KEGG as well as GSEA exhibited that the signaling pathway CTSA is involved in such as Lysosome, Oxidative phosphorylation, Metabolic pathways, etc., and should be further investigated in the future work. We further constructed a PPI network of CTSA co-expressed genes and screened out several genes including FTL, GRN, NPC2, HEXB, and PTGES2, which interact with CTSA. To the best of our knowledge, genes with similar expression patterns may be functionally related or even similar. The results of the Kaplan–Meier plotters database revealed that the high expression of these co-expressed gene mRNAs was related to the poor OS of HCC patients. Therefore, all our results indicate that CTSA may as an oncogene in the process of HCC tumorigenesis.

To gain more insight into the role of CTSA in HCC, we further queried its genetic alteration in a cohort of 359 LIHC patients in the cBioPortal database. The results showed that approximately 7% of CTSA exhibited the alterations, and these alterations were significantly related to poor OS. We further explored the reasons for this genetic alteration. From the PPI network, we screened out 5 co-expressed genes that interact with CTSA, and the high expression of these co-expressed genes was associated with poor OS of HCC. It is reasonable to assume that these co-expressed genes have an impact on the CTSA alterations and may play an important role in the tumorigenesis and development of HCC.

## Conclusion

The mRNA and protein expression level of CTSA in HCC tissues was significantly higher than adjacent normal liver tissues. High CTSA expression level was associated with poor clinical outcomes of HCC patients. CTSA can be used as a biomarker of the prognostic value of HCC. CTSA may as an oncogene that regulated tumorigenesis and development through influencing pathways such as lysosome, oxidative phosphorylation, and metabolic pathways, etc. Postoperative CTSA IHC examination can help predict the recurrence and prognosis of HCC patients.

## Data Availability

All datasets generated for this study are available within the article.
